# Supplementary material for: Can we use antipredator behavior theory to predict wildlife responses to high-speed vehicles?
Source: PLoS One. 2022 May 12;17(5):e0267774. doi: 10.1371/journal.pone.0267774 (PMC9098083; doi:10.1371/journal.pone.0267774)

**Supplementary Material**

S1: *Overview of model hypothesis, predictions, and assumptions*

1. Economic escape model (Ydenberg and Dill 1986)

Hypothesis:

Animals escape from a predator at the distance where the cost of not fleeing and the cost of fleeing curves intersect because that FID minimizes the reduction in future fitness.

Predictions:

- As the cost of not fleeing decreases, the slope of the line increases (the line becomes steeper), and the optimal FID decreases. A decrease in the cost of not fleeing is due to a decrease in predation risk.
- As the cost of not fleeing increases, the slope of the line decreases (the line becomes shallower), and the optimal FID increases. An increase in the cost of not fleeing is due to an increase in predation risk.
- As the cost of fleeing increases, the slope increases, and the optimal FID becomes shorter. An increase in the cost of fleeing is due to an increase in opportunities available (e.g., an increase in food abundance) to the animal.
- As the cost of fleeing decreases, the slope decreases, and the optimal FID becomes longer. A decrease in the cost of fleeing is due to a decrease in opportunities available (e.g., a decrease in food abundance) to the animal.

Assumptions:

- The predator is approaching an immobile animal
- Constraints Envelope: 0<FID<SD
- The model assumes animals monitor the approaching predator
  - Implicitly the model assumes that the animal has detected the predator
  - Animals do not escape immediately upon detection
- The intercept for the cost of not fleeing curve is the maximum possible loss of future fitness by the animal (Cooper and Frederick 2007).
- The intercept for the cost of fleeing is assumed to be 0. Both intercepts are fixed during the encounter (Cooper and Frederick, 2007).
- Both functions are monotonic, that the cost of not fleeing does not increase with an increase in distance, and the cost of fleeing does not decrease with an increase in distance (Cooper and Vitt, 2002).
  - The shape of both curves are unknown

Equations:

*F_0_* : The intercept for the cost of not fleeing curve,

*r* : The slope of the cost of not fleeing curve,

*s* : The slope of the cost of fleeing curve

*c* : The coefficient in the exponential equation for the cost of not fleeing curve that relates risk to distance.

Equation 1(Linear Cost of Not fleeing Curve): *R(d) = -r(d) + F_0_*

Equation 2 (Exponential Cost of Not fleeing Curve): *R(d) = F_0_ e^-cd^*

Equation 3 (Linear Cost of fleeing Curve): *S(d) = s(d)*

Equation 4 represents an additive relationship for the cost of fleeing and the cost of not fleeing and Equation 5 represents a multiplicative relationship.

Equation 4 (Additive Relationship): *F(d) =* [*F_0_-r(d)*] + [*s(d)*]

Equation 5 (Multiplicative Relationship): *F(d) =* [*F_0_-r(d)*] * [*s(d)*]

Figure S.1. A prey’s future fitness as a function of distance from the predator if the cost of fleeing and the cost of not fleeing are either additive or multiplicative. The x-axis is the distance between an immobile prey and an approaching predator. The black solid curve represents the prey’s future fitness as the result of its FID. F_0_ is the y-intercept and it represents the maximum possible loss of fitness if the prey comes into contact with the predator at distance 0. $s$and $r$ respectively represent the slope of the cost of fleeing and the cost of not fleeing. In panels a, b, and c the relationship between the cost of fleeing and the cost of remaining is additive. a) If the slopes are equal *(s=r)* than there is no predicted FID . b) if the slope for the cost of not fleeing is greater than the slope for the cost of fleeing *(s<r)*  the predicted FID should equal SD. c) if the slope for the cost of fleeing is greater than the cost of not fleeing *(s>r)* the predicted FID should equal 0. Panel d) the relationship between the cost of feeling and the cost of not fleeing is multiplicative, which has a single peak in fitness whose x-coordinate corresponds to the intersection of the curves, the predicted FID.

*
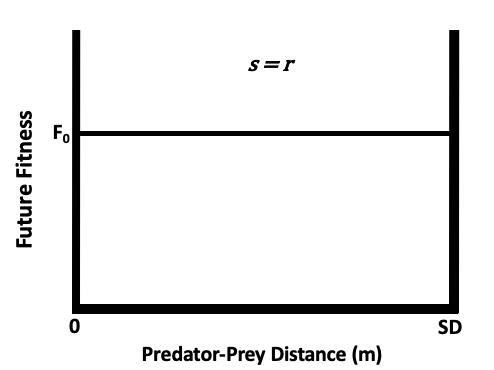
*

*
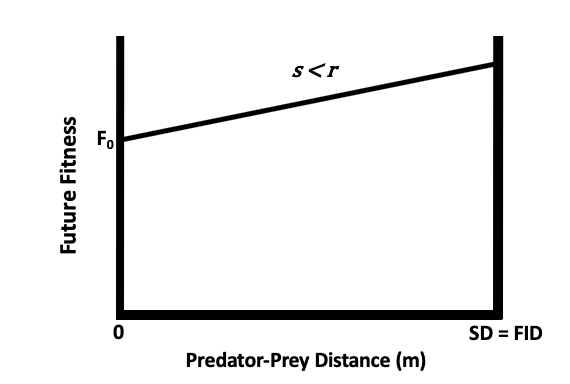

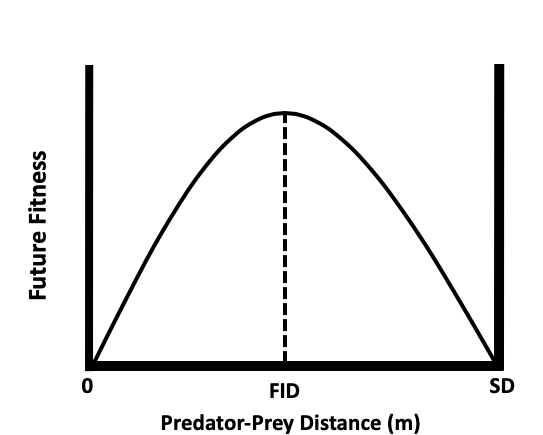

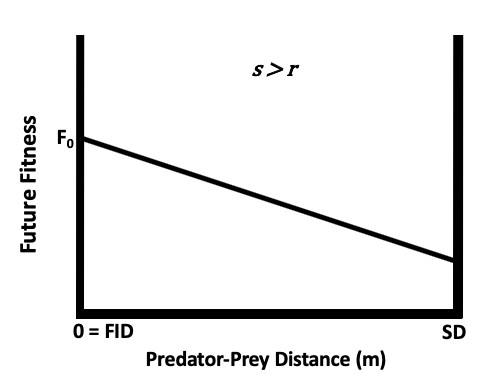
*

a)

c)

d)

b)

2.Blumstein’s economic escape model (Blumstein 2003)

Hypothesis:

Animals either escape immediately, do not escape, or monitor the predator and escape at the intersection of the two cost curves (the cost of not fleeing and the cost of fleeing) because they either perceive a maximum risk (zone I), a minimum amount of risk (zone III) or there is an intermediate level of risk and they escape at the distance which minimizes the reduction in future fitness (zone II).

Predictions:

- Animals do not escape if a predator is further away than d_max_.
- Animals escape immediately if a predator is detected at d_min_ or closer.
- In zone II, as the cost of not fleeing decreases, the slope of the line increases (The line becomes steeper), and the optimal FID decreases. A decrease in the cost of not fleeing is due to a decrease in predation risk.
- In zone II, as the cost of not fleeing increases, the slope of the line decreases (The line becomes shallower), and the optimal FID increases. An increase in the cost of not fleeing is due to an increase in predation risk.
- In zone II, as the cost of fleeing increases, the slope increases, and the optimal FID becomes shorter. An increase in the cost of fleeing is due to an increase in opportunities available (e.g., an increase in food abundance) to the animal.
- In zone II, as the cost of fleeing decreases, the slope decreases, and the optimal FID becomes longer. A decrease in the cost of fleeing is due to a decrease in opportunities available (e.g., a decrease in food abundance) to the animal.

Assumptions:

- The predator is approaching an immobile animal
- Possible range of FID predictions and definitions of each zone:
- Zone I: *0 < d < d_min_*
- Zone II: *d_min_* < *FID* < *d_max_*
- Zone III*: d > d_max_*
- The model assumes animals monitor the approaching predator
  - Implicitly the model assumes that the animal has detected the predator
  - Animals do not escape immediately upon detection
- Both functions are monotonic, that the cost of not fleeing does not increase with an increase in distance, and the cost of fleeing does not decrease with an increase in distance (Cooper and Vitt, 2002).
  - The shape of the curves is unknown

Equations:

Equations 1-3 are only applicable in Zone II and the distance is constraint between d_min_ and d_max_ (See section economic escape model).

3. Optimal escape model (Cooper and Frederick 2007)

Note: There are two versions of this model: 1) prey completely lose all fitness benefits when captured by the predator, and 2) an animal retains some fitness benefits despite being captured by a predator (Cooper & Frederick 2007). For our review we have focused on version 1) because it is unlikely that an animal retains any fitness benefits if it dies from a vehicle collision.

Hypothesis:

Animals obtain benefits by delaying escape which is offset by the metabolic cost of escaping. Animals weight benefits to their fitness by the decreasing probability of survival as the predator approaches closer. Animals escape at the distance which optimizes fitness because elsewise if the animal escapes before or after the optimal FID than after the encounter the animal will be left with a suboptimal fitness relative to other individuals within a population.

Predictions:

Table S.1. Qualitative differences in the predictions of the optimal escape model.

| Parameter | Symbol | | FID | Assumption |
| --- | --- | --- | --- | --- |
| Initial Fitness | | *F_0_* $\uparrow$ | $\uparrow$ |  |
| Maximum Benefit | | *B**$\uparrow$ | $\uparrow$ |  |
| The rate at an animal obtains benefits | | *n*$\uparrow$ | $\uparrow$ | n>>1, Increases asymptotically |
|  |  | *n* $\uparrow$ | $\downarrow$ | 0<n<1 |
| Escape Function | | *E(d)* $\uparrow$ | $\downarrow$ |  |
| The coefficient relating escape cost to distance | | *m* $\uparrow$ | $\downarrow$ | m>0 |
| The exponent relating escape cost to distance | | *f* $\uparrow$ | $\downarrow$ |  |
| The probability of survival function | | *P_s_(d)* $\uparrow$ | $\downarrow$ |  |
| The coefficient which relates the probability of survival to distance | | *c* $\uparrow$ | $\downarrow$ | \|c\| |

1. Assumptions:

- Animals act optimally
- The encounter begins when the animal detects the predator (detection distance = DD)
- Constraint Envelope: 0< FID < DD
- An animal’s future fitness is 0 if it does not escape (*FID=0*) (Cooper and Frederick 2007).
- The animal’s initial fitness is fixed at the beginning of the encounter (DD) (ie., F_0_ is not a function of distance
- The escape cost is a one-time energetic cost caused by the movement associated with escape
- At distance 0, when the predator captures the prey, the escape cost is 0 because the animal did not escape
- Probability of survival is likely to be lower at shorter distances and higher at longer distances

1. Equations:

*F(d)*: The optimal FID for the animal’s fitness

*F_0_*: Initial Fitness

*B(d)*: Benefits as a function of distance

*E(d)*: Escape cost (the energetic cost of escaping) as a function of distance

*P_s_ (d)*: Probability of Survival as a function of distance

Equation 1: $F\left( d \right)=\left[ F_{0}+B\left( d \right)-E\left( d \right) \right]Ps(d)$

*F_0_* : Initial Fitness

*B**: Maximum benefit

DD : detection distance

*n* : the rate an animal can obtain benefits

*f* : coefficient relating the escape cost to distance

*m* : exponent relating the escape cost to distance

*c* : *t*he coefficient for the probability of survival as a function of distance

Equation 2: $F\left( d \right)=\left[ F_{0}+B^{*} [{(1-\frac{d}{d_{d}})}^{n}]\text{ }-{f(d)}^{m} \right]$*$(1-e^{-cd})$

4. The perceptual limits hypothesis (Quinn and Cresswell 2005)

Hypothesis:

Animals escape when they detect a predator and because detection is a function of the animal’s sensory systems, escape is limited by the perceptual abilities of that species.

Predictions:

- Animals escape immediately upon detection
- Species with a more constrained sensory system detect objects at closer distances and thus have a shorter FID.
- Species with a sensory system that allows them to detect objects further away will have a longer FID.

Assumptions:

- FID = DD
- SD > DD
- SD > FID
- There is no assessment time or alert distance
- The difference between SD & DD is a function of that species’ sensory constraint
- Additional Assumptions Related to FID estimates based on visual acuity.
- The object occupies one degree of a species visual field at the detection distance
- The animal will be able to identify the threat at the moment of detection
- The animal views the approaching threat monocularly
- Viewing occurs under optimal ambient light

Equation: Based on a species visual acuity

DD : detection distance

*r* : is the radius of the object

$\alpha$: is the inverse of that species spatial resolving power (eye size and cell density) (Pettigrew et al. 1988 & Tyrell et al. 2013)

$Equation 1: d_{d}=\frac{r}{\tan( \frac{\alpha}{2} )}$


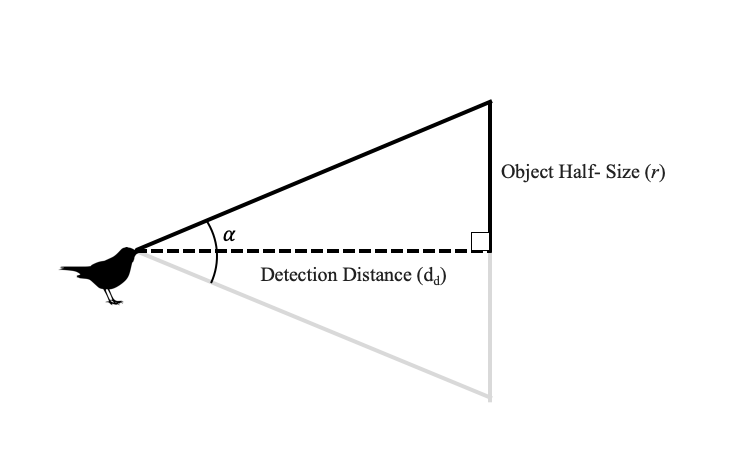
Figure S.2. A prey’s detection distance can be estimate using the tangent formula (tanθ = $\frac{Opposite}{adjacent}$) for a theoretical right triangle between the approaching object and the viewer. A prey’s visual acuity ($a$) (cycles per degree) sets the distance when the prey can see the approaching object at 1 degree in the field of view. The opposite side is the objects half-size (*r*) and the adjacent side is the predicted detection distance (DD).

5. The flush early and avoid the rush hypothesis (Blumstein 2010)

Hypothesis:

The FEAR hypothesis argues that animals tend to escape early to minimize the cost of monitoring an approaching predator because they have limited attention; thus, allocating attention towards monitoring the predator diverts attention away from other potential fitness enhancing activities, such as foraging, mating, etc.

Predictions:

FID and alert distance (AD) are positively correlated because animals escape soon after detection (AD).

Assumptions:

- Possible range of FID predictions: 0<FID<AD<SD
- The maximum slope is 1 when FID = AD
- The intercept goes through 0 because If AD= 0 then FID = 0
- The model assumes alert distance is a proxy of detection distance
- An animal has not detected the predator prior to its alert distance
- The relationship between FID and AD is linear

Equation:

Equation 1: *Phi-Index:* $\phi=1- \frac{\sum_{i=0}^{n} \frac{({AD}_{i}-{FID}_{i})}{{AD}_{i}}}{n}$

**If your data supports the F.E.A.R. Hypothesis the phi-index, that is* $\phi$ *is greater than 0.5 and the AD and FID relationship is significant*

$\beta$: the slope predicted by a regression analysis

The intercept is forced through the origin (assuming FID = 0 when AD = 0)

AD: the mean alert distance

Equation 2: *FID*$=\beta*AD$

Note: We compared$\phi$ , a proxy for the slope of an AD vs. FID relationship, with the estimated slope from a linear model for each speed treatment. For each linear model of an AD vs FID relationship we assumed the intercept of the model was 0 in accordance with the FEAR hypothesis. Both $\phi$ and the estimated value of the slope from a linear model were positively correlated with a slope of 1.23. Therefore, because of the phi-index tendency to overestimate the value of the observed slope of the AD vs. FID relationship for the DeVault et al. 2015 dataset. We decided to generate the FID predictions using the observed slope.

Predicted FID based on DeVault et al. 2015 data

Figure S.3. The relationship between the predicted FID (m) and vehicle approach according to the FEAR hypothesis. The grey shading represents the 95 percent confidence interval. The mean FID across speed treatments was 14.53 m and the mean $\phi$ was 0.722.


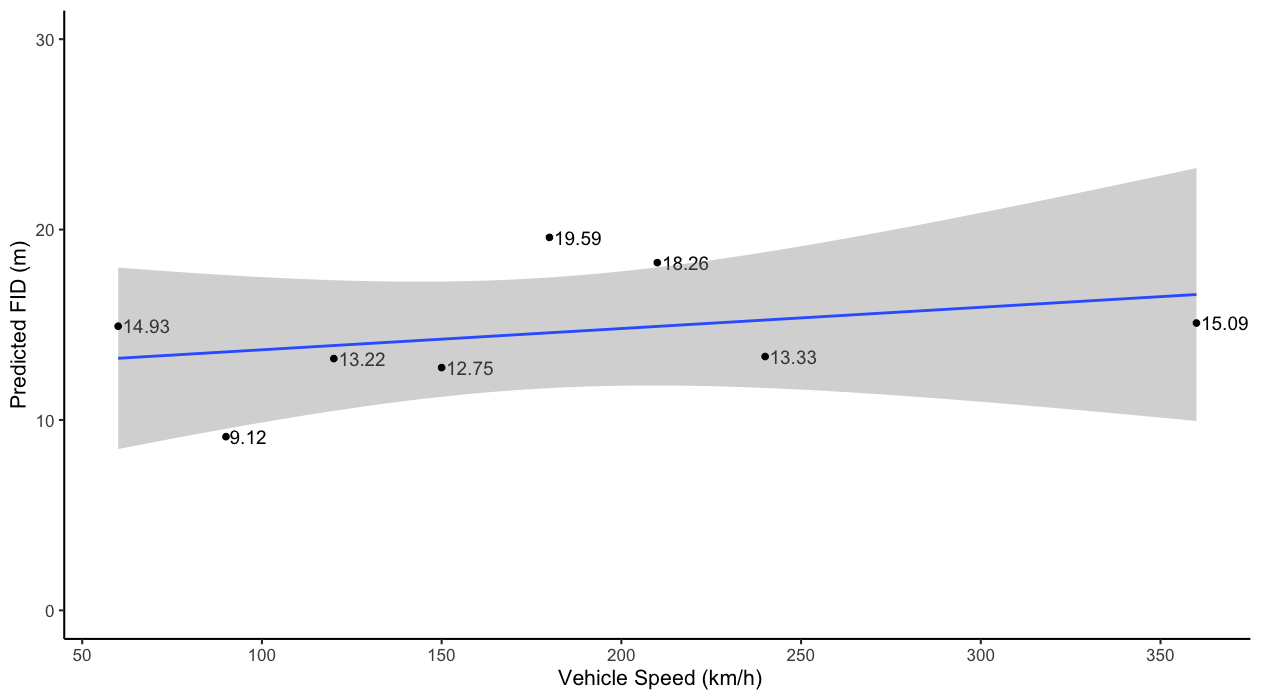


6. The looming stimulus hypothesis (Schiff et al. 1962, Sun and Frost 1998)

Hypothesis:

Animals escape because neurons activate when the ratio of the visual angle, $\theta\left( t \right),$and the rate at which the visual angle expands, $\theta$’($t$), $\tau$, decreases to a critical threshold triggering an escape response.

Predictions:

Animals escape from an approaching object when $\tau$, ($\frac{\theta\text{(t)}}{\theta\text{’}\text{(t)}}$), reaches a critical threshold.

Assumptions:

- A looming Stimulus is based on monocular vision
- Time represents seconds from collision and at time 0, the object (ie., vehicle or predator) collides with the animal
- Visual angle at time to collision = 180 degrees (based on monocular vision)
- At the neuron’s peak firing rates, some threshold value of critical ratio ($\frac{\theta\text{(t)}}{\theta\text{’}\text{(t)}}$), we assume the animal escapes

Equations:

$\theta_{threshold\left( t \right)}$: The threshold visual angle at which the animal escapes

*v* : velocity, the velocity at which an object approaches a vehicle is assumed to be constant.

*r* : the half size of the object

$\delta$ : the physiological delay between when the neuron begins to fire and its peak firing rate

$$Equation 1: \theta_{threshold\left( t \right)}=2*{tan}^{-1}\left( \frac{r}{v*{TTC}_{Flight}} \right)$$

Equation 2:${\theta^{'}}_{threshold(t)}=\frac{1}{\frac{v}{r*2}*{\text{TTC}\text{Flight}}^{2}+ \frac{r*2}{4*v}}$

Yan JJ, Lorv B, Li H, Sun HJ. Visual processing of the impending collision of a looming object: Time to collision revisited. Journal of vision. 2011 Oct 1;11(12):7-.

Equation 3:$FID=\left( \frac{r}{\tan\left( \frac{\theta_{threshold\left( t \right)}}{2} \right)} \right)-\left( v*\delta\right)$

Predicted FID based on DeVault et al. data

Figure S.4. The relationship between the predicted FID (m) and vehicle approach speed (km/h) according to the looming stimulus hypothesis. The grey shading represents the 95 percent confidence interval. The mean FID across speed treatments was 30.03 m.


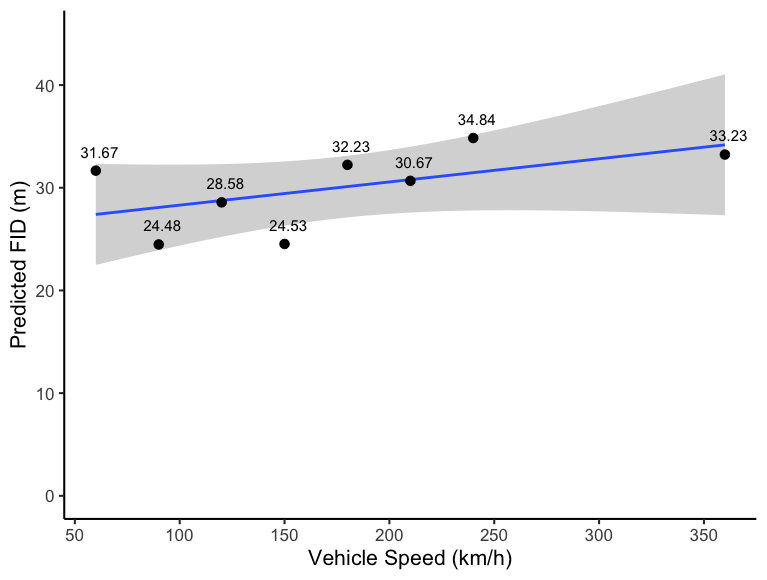


7. Visual cue model (Javurkova et al. 2012)

Hypothesis:

Animals escape in response to a change in the ratio between perceived profile size of an approaching object and distance away from the object. When this ratio exceeds a species specific threshold triggering the animal escapes because animals rely on perceived object size to determine distance away from an object similar to the looming stimulus hypothesis.

Predictions:

Animals escape at the distance where the ratio between an approaching objects profile size and distance exceeds a species specific threshold.

Assumptions:

- Animals escape in response to changes in the ratio of the approaching objects diameter to distance from the object
- Animals view the approaching threat at two distinct distances and only escape after that second viewing distance
- The animal has detected the predator at it first viewing distance
- Predator approach speed is constant
- Escape behavior varies between individuals

Equations:

$A_{0} :$Actual size of the predator

$\Delta A$: Change in predator profile size

*c* : The proportion of the visual signal concealed by vegetation

*d* : Distance from the predator

$\alpha$: The approach angle of the predator

Note: equation 5 is used to predict FID with the bisection method

Equation 1: ${A'}_{d}=\frac{A_{0}}{d^{2}}$

Equation 2: ${A'}_{d'}=\frac{A_{0}}{{(d-\Delta'd)}^{2}}$

Equation 3: $\Delta A=\left( 1-c \right)({A^{'}}_{d^{'}}-{A^{'}}_{d})$

Equation 4: $\Delta A=\left( 1-c \right)A_{0}\left( \frac{1}{(d-\Delta dcos \alpha^{2}+\Delta^{2}d{sin}^{2}\alpha}-\frac{1}{d^{2}} \right)$

$$Equation 5: 0=\left( 1-c \right)\left( \frac{1}{(FID-\Delta dcos \alpha^{2}+\Delta^{2}d{sin}^{2}\alpha}-\frac{1}{{FID}^{2}} \right)-\frac{\Delta A}{A_{0}}$$

Predicted FID based on DeVault et al. 2015 data

It is important to note that the predictions for this model are based on the mean AD for each speed treatment in DeVault et al. 2015. The predicted FID for this model is based on how perceived profile size changes with distance and does not incorporate any parameters which are sensitive to approach speed. As a result we know that the visual cue model is not sensitive to approach speed. The difference in predicted FID displayed below are the result of difference in the empirically observed AD. We present this figure to demonstrate the model’s abilities to make different quantitative predictions and the variation within those predictions.

Figure S.5. The relationship between the predicted FID (m) and vehicle approach speed (km/h) according to the visual cue model. The grey shading represents the 95 percent confidence interval. The mean FID across speed treatments was 19.25 m.


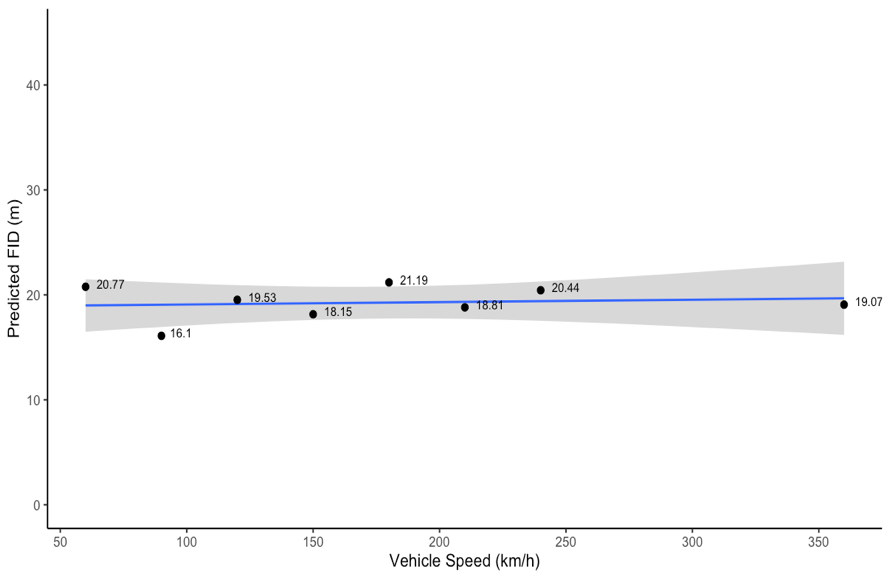


8. Bayesian Optimal Escape Model (Sutton and O’Dwyer 2018)

Hypothesis:

Bayesian optimal escape model, animals escape (i.e., FID) at the point where the energetic cost of fleeing is equal to or less than the perceived energetic cost of remaining, which is weighted by their perceived probability of attack. The perceived probability of attack, is subject to the animal’s behavioral bias and information associated with the approaching predator.

Predictions:

Animals escape when the perceived cost of remaining is greater than the energetic cost of fleeing.

Assumptions:

- Detection is equal to alert distance
- Information about the risk associated with the predator *f(x)*(see below), is received in the same for all individuals
- The parameter α represents the influence of all prior experiences that might bias an individual’s perception of risk.
- is between 0 and 1
- Risk factors related to the perceived probability of attack are considered independently from each other

Equations:

$\beta$: The energetic cost of fleeing

$E:$The perceived energetic cost of remaining

$P\left( A | X \right):$ The perceived probability of attack given some information with regards to risk

$\alpha$: An individual specific coefficient that represents individual behavioral bias for an animal. Potential factors which may bias the perceived probability of attack are past experience, habituation, or an individual’s personality.

$f(x)$: A function for the information animals use to assess risk for the approaching predator

$r\left( t \right):$ predator-prey distance

*AD* : Alert Distance

$v_{r}$: The predator’s approach speed

$v_{max}$: The maximum velocity of the species

$$Equation 1: \beta\leq E \times P(A|X)$$

Equation 2: $P(A|X)$ $= (\frac{\alpha f(x)}{\alpha f\left( x \right)+\left( 1-f\left( x \right) \right)(1-a)}$)

$$Equation 3: f_{1}\left( r \right)=P\left( A \right|r, \alpha=0.5)=\left\{ \begin{aligned} 1-\frac{r\left( t \right)}{AD} if r\left( t \right) \leq AD \\ 0 if r\left( t \right) >AD \end{aligned} \right.$$

$$Equation 4: f_{2}\left( r \right)=P\left( A \right|v_{r}, \alpha=0.5)=\left\{ \begin{aligned} \frac{v_{r}}{v_{max}} if v_{r} \leq v_{max} \\ 1 if v_{r} \leq v_{max} \end{aligned} \right.$$

Figure S.6. The predicted FID as vehicle approach speed increases (m/s) according to the Bayesian optimal escape model.. The grey shading represents the 95 percent confidence interval. The mean FID across speed treatments was 44.99 m.


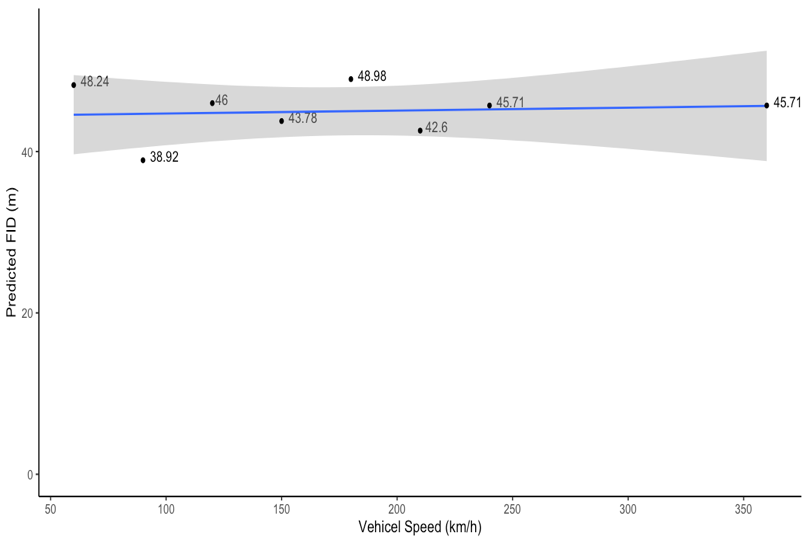

Supplement: S1 Appendix — (DOCX) [file pone.0267774.s001.docx]
