# Supplementary material for: Can we use antipredator behavior theory to predict wildlife responses to high-speed vehicles?
Source: PLoS One. 2022 May 12;17(5):e0267774. doi: 10.1371/journal.pone.0267774 (PMC9098083; doi:10.1371/journal.pone.0267774)
Supplement: S2 Appendix — (DOCX) [file pone.0267774.s002.docx]

S2: An overview of the economic escape model, Blumstein’s economic escape model, and the optimal escape model. The section discusses the limitations to generating quantitative predictions and sensitivity to speed for each model.

***Economic escape model***

1. Overview

The Economic escape model proposes an ultimate explanation because it assumes that the distance at which an animal escapes will affect fitness. Ydenberg and Dill’s (1986) _[18]_ graphical model represents a scenario in which an animal monitors an approaching predator and escapes at the distance which minimizes the cost to future fitness (S1.1). In the model, the interaction between predator and prey begins at the point where the predator starts its approach (SD). Therefore, the model assumes that at some distance (unspecified by the model, possibly >SD) the predator is detected and that the approach is monitored by the prey. The model posits that the decision of the animal to escape at a certain distance is a function of two cost curves: the cost of fleeing and the cost of not fleeing (Fig 1a). The cost of fleeing curve is associated with the opportunities an animal foregoes when fleeing from a predator (i.e., opportunity cost), and this curve increases with distance from the predator. If the animal escapes prematurely, it will forego a larger amount of benefits (i.e., foraging) than if it delays escape (Fig 1a – blue solid line) _[18]_.

Ydenberg and Dill (1986) _[18]_ portrayed the cost of fleeing curve as linear. The cost of not fleeing is associated with the risk of dying from the predator and its curve decreases with distance from the predator because the risk of dying decreases the farther away the animal is from the potential source of mortality (Fig 1a – red solid line). The model assumes that the intercept for the cost of not fleeing curve is the maximum possible loss of future fitness (i.e., dying), if the animal comes into contact with the predator (i.e., distance = 0) _[25]_. The original model portrayed the cost of not fleeing as nonlinear (i.e., exponential); however, the exact shape of the curve is unknown empirically, so we have represented it as linear (Fig 1a) for simplicity and following Cooper & Vitt (2002) _[67]_. The model predicts that the distance at which animals escape (i.e., FID) will be the intersection of the cost of not fleeing curve and the cost of fleeing curve because at this distance the reduction in future fitness will be minimized (Fig 1a – dotted black line). The exact relationship between FID and fitness is not specified; consequently, the shape of the curves are unknown _[25,67]_. The original graphical model does not provide a mathematical formulation for either curve; however, Cooper and Frederick (2007) _[25]_ suggested a mathematical formulation for an exponential or linear cost of not fleeing curves and a linear cost of fleeing curve (S1.1). Both cost curves are assumed to be monotonic _[67]_.

The economic escape model has been the theoretical cornerstone for decades of escape behavior research _[8]_. While this graphical model maintains heuristic value, there are several gaps that should be addressed. First, the model depicts the cost of fleeing and the cost of not fleeing curve as independent of each other, yet it is possible that characteristics of the approaching predator simultaneously affect both cost curves. For example, if an animal is approached by a predator at a fast speed not only is there an increase in risk perception (an increase in the cost of not fleeing), but simultaneously there is less time to take advantage of a given opportunity (a decrease in the cost of fleeing). This potential lack of independence of the two curves can affect its predictions. Second, the curves should not be represented as equivalent. If an animal fails to escape then the consequence (i.e., death) results in a loss of all future fitness. In comparison, the one-time cost of fleeing prematurely from a predator is most likely non-consequential at that moment in time (i.e., less foraging time), and assuming that refugia present less risk. In that scenario, we would expect the slope of the cost of fleeing curve to be small (i.e., close to zero) if not constant. Third, the two curves impact future fitness on two different time scales. The cost of not escaping impacts future fitness immediately if a prey is captured by a predator, but the realized impact of the cost of fleeing on future fitness is the result of all cumulative opportunities the animal has left behind, and relative to resources elsewhere. Animals that tend to have a longer FID over the course of a lifetime would likely have a reduced future fitness compared to animals with a shorter FID. Careful consideration of the misalignment in temporal scales of the two costs curves is critical for the ability of this model to generate quantitative predictions (see below).

2. Quantitative predictions

According to the economic escape model, the predicted FID will be at the intersection of the two cost curves. The relationship between the two curves should be either additive or multiplicative (S1.1). We can hypothetically explore the relationship between the two cost curves and fitness by either multiplying or adding the equations for the two cost curves put forth by Cooper and Frederick (2007) _[25]_ for this model. This was done as a mathematical exercise and not based on any empirical data. We explored which relationship between the two curves, if any, produces a fitness curve that peaks for a single FID distance (S1.1). When the two cost curves are added and the absolute values of the slopes are equal, fitness does not change with the distance to the predator (S1.1), leading to no optimal FID regardless of the intersection in the two cost curves. Alternatively, if we add the two cost curves and the absolute value of the slopes for the cost of not fleeing and the cost of fleeing are not equal, then the optimal FID is either 0 or FID is equal to the distance at which the predator initiates the approach (SD) (S1.1). Ideally, however, costs of remaining or fleeing should be expressed as likelihoods. Thus, only when the two curves are multiplied by each other is there a single peak in future fitness for a given predicted FID as a result of the two curves crossing (S1.1). The implication is that to generate quantitative predictions with this model the two cost curves should have a multiplicative relationship.

Unfortunately, the relationship between the cost curves and future fitness is unknown empirically. One possible set of proxies for the cost of not fleeing curve and the cost of fleeing is the probability of capture, or in the case of animal-vehicle interactions, the probability of collision with a vehicle and the probability of starvation based on FID _[68,69]_. Survivorship is a critical factor in future fitness; so the probability of capture makes a suitable proxy for the cost of not fleeing curve _[69]_. The cost of fleeing might have a wide range of fitness consequences as a result of missed foraging or missed mating opportunities _[18]_ . The economic escape model represents a single event and the interaction between an animal and an approaching vehicle is likely to happen on the order of seconds. Additionally, the decisions of animals to return to the spot where the interaction occurred can differ depending on whether the interaction was with a predator vs. a vehicle (i.e., shorter return times are expected with vehicles) or if the animal was missed by the vehicle or evaded (absent leaving the area) a collision without fitness-impacting effects. How these differences impact fitness is unclear empirically, which limits our ability to parameterize the model.

Overall, for the economic escape model to generate quantitative FID predictions we would need to parameterize both curves with empirical data. As discussed, generating a proxy for the cost of not fleeing curve is possible, but we have not found a suitable proxy for the cost of fleeing curve. Therefore, we could not generate quantitative predictions for this model in the context of animal-vehicle interactions.

3. Sensitivity to approach speed

Because we could not generate quantitative predictions for the economic escape model, we did not explore its sensitivity to speed from a quantitative point of view.

***Blumstein’s economic escape model***

1. Overview

Blumstein’s economic escape model _[24]_ attempts to provide an ultimate explanation because short-term escape decisions are linked to fitness. Blumstein’s model expands upon Ydenberg and Dill’s model _[18]_ by partitioning the graphical model into three different zones, instead of only one, based on the response to the approaching predator (S1.2). The model starts at the distance where the predator begins its approach towards the animal (SD). Blumstein’s escape model assumes that the detection of the predator could occur in any of the three zones, and where detection occurs results in different behavioral outcomes. In other words, detection distance (DD)≤SD. In zone I, animals perceive a maximum amount of risk, and if they detect the predator in this zone, they escape immediately (Fig 2a). In zone II, upon detection animals monitor the approaching predator and escape at the distance that minimizes the reduction in fitness as determined by the intersection of the two cost curves (the cost of not fleeing and the cost of fleeing; Fig 2a), following from the economic escape model _[18]_. In zone III, animals do not escape because either they have detected the predator and perceive a minimum amount of risk or have not detected the predator at all (Fig 2a). Zones I and II and zones II and III are bounded by two threshold distances (the minimum distance, d_min_; and the maximum distance, d_max_; respectively) that establish where the animal will either escape immediately if a predator is detected closer than some minimum threshold distance or will not escape at all when a predator is beyond that maximum distance. Although the intersection of the cost curves determines the FID in zone II, as indicated for the economic escape model, animals escape immediately if the predator is detected closer than the predicted FID.

2. Quantitative predictions

The logic of Blumstein’s economic escape model is similar to the economic escape model (see above). Zone II is limited to qualitative predictions because there is not empirical evidence on how the cost of fleeing is related to fitness, as discussed before. To generate quantitative predictions, the cost of fleeing and the cost of not fleeing curves should have a multiplicative relationship with each other, must be independent of each other, and have costs that can be comparable temporally. Consequently, it is not possible to generate quantitative FID predictions in the context of animal-vehicle interactions with this model.

3. Sensitivity to approach speed

Because we could not generate quantitative predictions for the economic escape model, we did not explore its sensitivity to speed from a quantitative point of view.

***Optimal escape model***

1. Overview

The optimal escape model is different from the prior two models in that animals can enhance their fitness by optimizing the decision as to when to escape from a predator, as opposed to minimizing the cost to future fitness _[25]_. The optimal escape model can be considered an ultimate explanation for escape distance because escape decisions are expected to affect fitness. The model begins at the distance where the animal detects the approaching predator (DD). Future fitness is determined by how initial fitness is affected by three functions that vary relative to the distance from the predator: (1) benefits obtained from delaying escape (i.e., more time spent foraging instead of escaping), (2) the escape cost (i.e., the metabolic costs associated with escaping), and (3) the probability of survival (i.e., avoiding being caught as the predator approaches). Each function is made up of several parameters that generate a predicted FID (S1.3) _[25]_. An animal can enhance fitness by delaying escape (i.e., continue foraging) and allowing the predator to approach closer (Fig 2b). These benefits are offset by a one-time cost of escape, and ultimately weighted by the decreasing probability of survival as the predator gets closer (Fig 2b) _[25]_. Initial fitness remains constant regardless of the distance at which the animal decides to escape (S1.3). The model predicts that the animal will escape at the distance that optimizes fitness relative to all other distances (Fig 2c; S1.3) _[25]_.

2. Quantitative predictions

For the optimal escape model to make quantitative predictions about escape distance, empirical estimates of the fitness benefits obtained from delaying escape and the escape costs relative to initial fitness are necessary. However, we are unaware of any such empirical evidence over the span of a few seconds. Consequently, we could not generate quantitative predictions in the context of animal-vehicle collisions for the optimal escape model.

3. Sensitivity to approach speed

Because we could not generate quantitative predictions for the economic escape model, we did not explore its sensitivity to speed from a quantitative point of view.

1. Cooper Jr WE, Vitt LJ. Optimal escape and emergence theories. Comments® on Theoretical Biology. 2002 Sep;7(5):283-94.
2. Lagos PA, Meier A, Tolhuysen LO, Castro RA, Bozinovic F, Ebensperger LA. Flight initiation distance is differentially sensitive to the costs of staying and leaving food patches in a small-mammal prey. Canadian Journal of Zoology. 2009 Nov;87(11):1016-23.
3. Lagos PA, Ebensperger LA, Herberstein ME. A quantitative test of the ‘economic’and ‘optimal’models of escape behaviour. Animal behaviour. 2014 Nov 1;97:221-7.

Figure S.7. a) Economic escape model is a graphical model. The x-axis is the distance between an animal and an approaching threat (i.e., predator-prey distance). The red curve represents cost of not fleeing, R(d), and the blue curve represents the cost of fleeing, S(d). The x-coordinate where the two curves intersect is the predicted FID for the animal. b) As speed increases, the cost of not fleeing curve becomes more shallow and an animal’s predicted FID increases (R2(d)). As risk increases the absolute value of the slope for the cost of not fleeing curve approaches 0. c) If the two curves do intersect than FID becomes limited by the cost of fleeing.


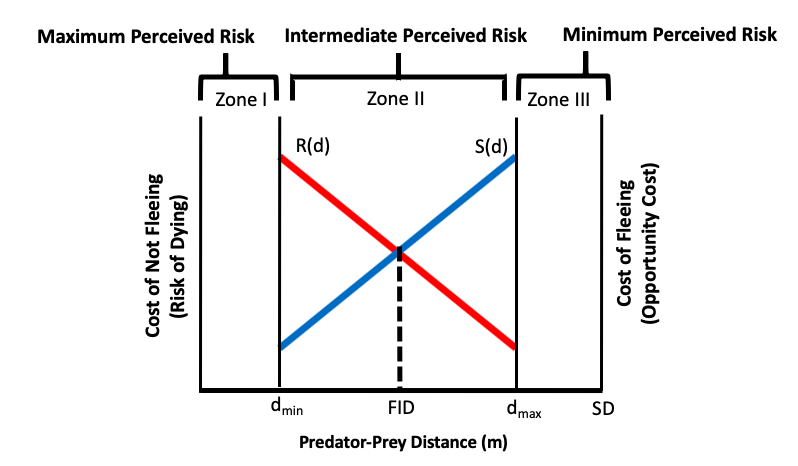


b)

a)

*
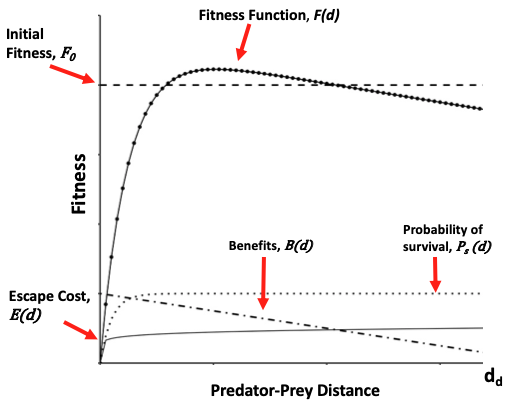
*

DD

*
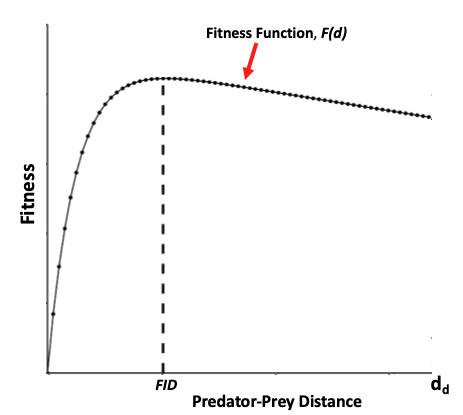
*

c )
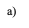
)

Figure S.8 .a) Graphical representation of Blumstein’s economic escape model. The x-axis is the distance between an immobile animal and an approaching threat (i.e., predator-prey distance). The red curve represents the cost of not fleeing, R(d), and the blue curve represents the cost of fleeing, S(d). a) In the original formulation Zone I, is from 0 to *d_min_* and an animal always flee if a threat is detected. Zone II is from *d_min_* to *d_max_* and the predicted FID is determined by the animals assessment of the cost of not fleeing and the cost of fleeing. Zone II is similar to Economic escape model. Zone III is any distance greater than d_max_ and the animal does not respond to threats in this zone. b) Optimal escape model offers explicit mathematical formulations for an animal’s escape behavior. The x-axis is the distance between an immobile animal and an approaching threat. The interaction begins with the animal’s detection distance (DD)*.* In optimal escape model an animal’s fitness, *F(d*), is determined by how initial Fitness ,(*F_0_*), the benefits gained during a threat’s approach, *B(d*), and the escape cost ,*E(d*), multiplied by the probability of survival, *P_s_(d*), affect an animal’s initial fitness (*F_0_*). Fitness increases as the benefits functions increases at shorter distances away from the threat. Fitness then decreases as the probability of survival decreases at very close distances to the threat. Escape cost is a one-time cost the prey pays when it flees. Initial fitness does not change with distance. c) Animal escape (FID) at the peak of the fitness function (*F(d*)) in the optimal escape model.

DD


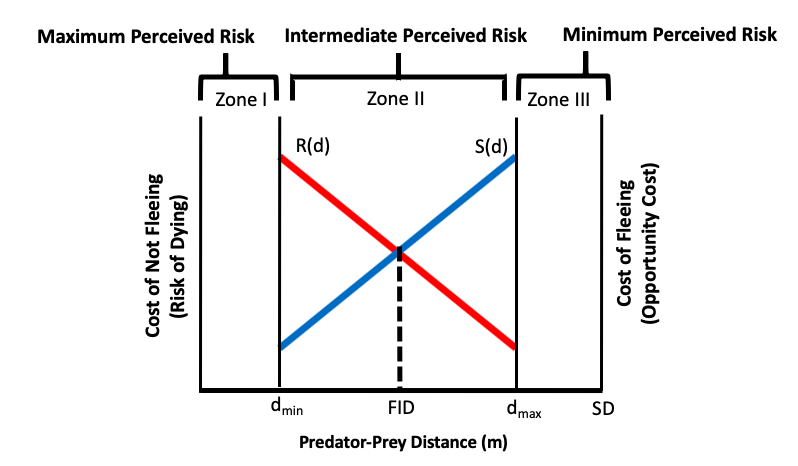


*
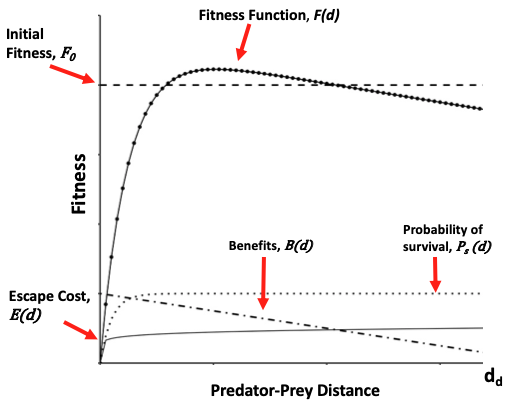
*

b)

a)

DD

*
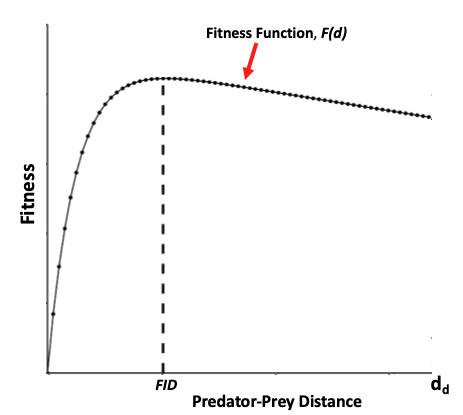
*

c
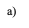
)

DD
