# Supplementary material for: Can we use antipredator behavior theory to predict wildlife responses to high-speed vehicles?
Source: PLoS One. 2022 May 12;17(5):e0267774. doi: 10.1371/journal.pone.0267774 (PMC9098083; doi:10.1371/journal.pone.0267774)
Supplement: S3 Appendix — (DOCX) [file pone.0267774.s003.docx]

S3: *Established distributions of FID, AD, slope and intercept speed and FID relationship*

Below are the 9 papers used to generate the observed distributions of FID, AD, and the slope and intercept speed and FID relationship based on our search of the literature.

Bernard, G. E., van Dongen, W. F., Guay, P. J., Symonds, M. R., Robinson, R. W., & Weston, M. A. (2018). Bicycles evoke longer flight-initiation distances and higher intensity escape behaviour of some birds in parks compared with pedestrians. *Landscape and urban planning*, *178*, 276-280.

DeVault, T. L., Blackwell, B. F., Seamans, T. W., Lima, S. L., & Fernández-Juricic, E. (2014). Effects of vehicle speed on flight initiation by turkey vultures: implications for bird-vehicle collisions. PLoS One, 9(2), e87944.

DeVault, T. L., Blackwell, B. F., Seamans, T. W., Lima, S. L., & Fernández-Juricic, E. (2015). Speed kills: Ineffective avian escape responses to oncoming vehicles. Proceedings of the Royal Society B: Biological Sciences, 282(1801).

DeVault, T. L., Seamans, T. W., Blackwell, B. F., Lima, S. L., & Fernández-Juricic, E. (2017). Individual variation in avian avoidance behaviours in response to repeated, simulated vehicle approach. *Canadian Journal of Zoology*, *96*(5), 441-446.

Guay, P. J., McLeod, E. M., Taysom, A. J., & Weston, M. A. (2014). Are vehicles' mobile bird hides'?: A test of the hypothesis that'cars cause less disturbance'. *Victorian Naturalist, The*, *131*(4), 150-156.

Legagneux, P., & Ducatez, S. (2013). European birds adjust their flight initiation distance to road speed limits. *Biology letters*, *9*(5), 20130417.

- We only used species which were approached directly on road ways

Lethlean, H., Van Dongen, W. F., Kostoglou, K., Guay, P. J., & Weston, M. A. (2017). Joggers cause greater avian disturbance than walkers. *Landscape and Urban Planning*, *159*, 42-47.

McLeod, E. M., Guay, P. J., Taysom, A. J., Robinson, R. W., & Weston, M. A. (2013). Buses, cars, bicycles and walkers: the influence of the type of human transport on the flight responses of waterbirds. *PLoS One*, *8*(12), e82008.

Figure S.9. The established distributions of alert distance, flight initiation distance, slope, and intercept of the FID and approach speed relationship. a) The distribution of mean alert distances of 50 species; AD values ranged from 9.9 m to 63.7 m, with the mean (± SD) AD being 49.55 ± 10.51 m. b) The distribution of mean flight initiation distances of 50 species; FID values ranged from 5.55 m to 128.38 m, with mean (± SD) FID being 43.97 ± 31.08 m. c) The distribution of the slopes of the relationship between FID vs. speed ranged from -37.46 to 32.33 (mean ± SD, -2.12 ± 10.95). d) The distribution of intercepts for the relationship between FID vs. speed ranged from -75.70 to 128.4 (mean ± SD, 42.98 ± 46.27).


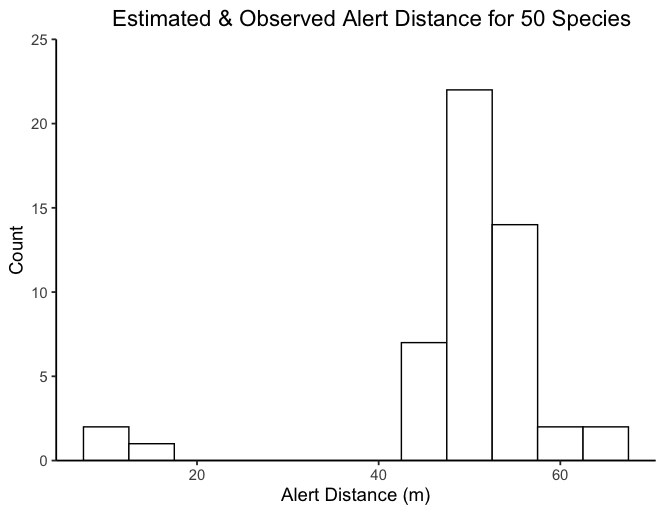

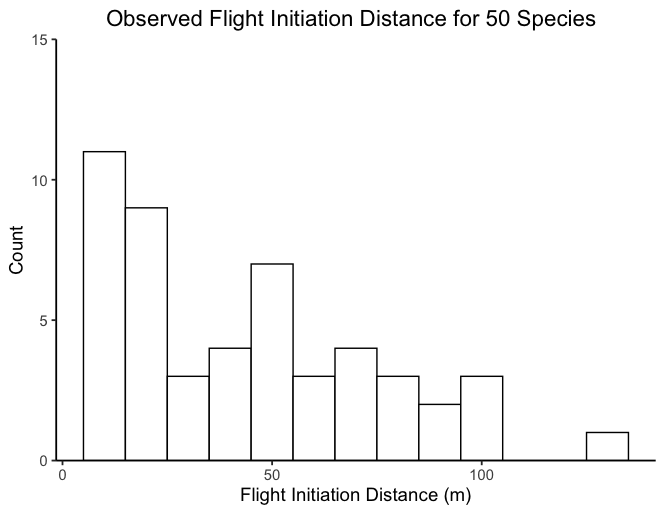


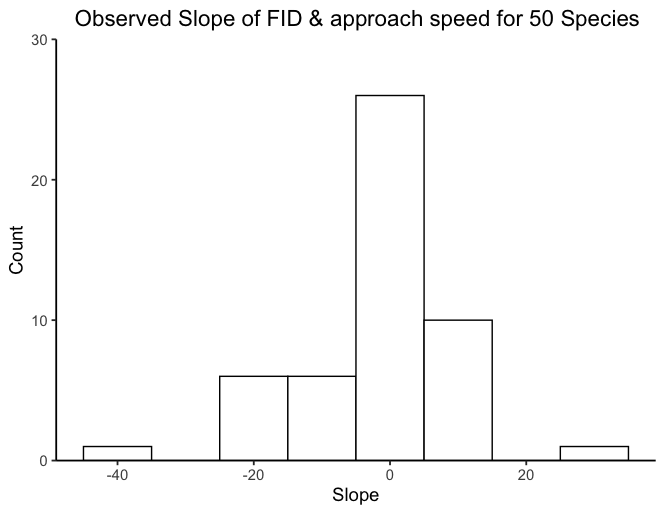

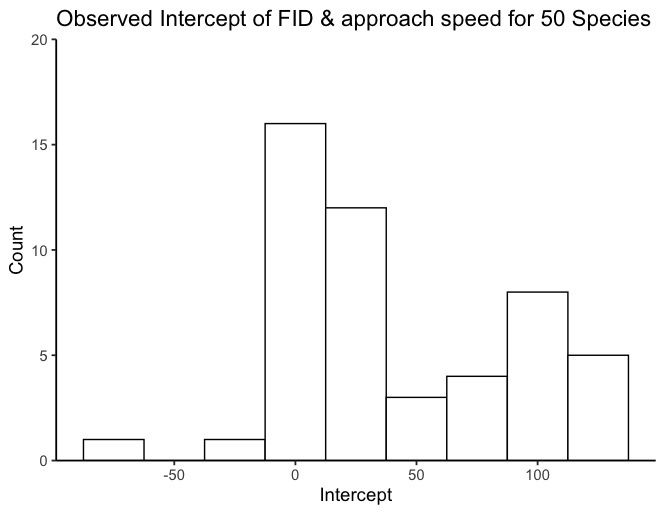


Table S.2. The predicted slope between FID and approach speed for each species. The predicted intercept between FID and approach speed for each species. FID values are the mean FID of all the empirically observed FIDs. We obtained the mean AD for only two of the 50 species we had approach speed and FID data for. When AD data was not reported in the 9 studies we identified in our literature search (48 species), we used AD data reported in other papers for that specific species (an additional 2 species) (Fernandez-Juricic et al. 2001, Blumstein et al. 2004, ). If a species had not reported AD, we estimated it based on the species mean body mass (46 species) (Appendix 1..) (following Blumstein et al. 2005). Below * next to the AD value denotes empirically reported body masses.

| *Species Name* | *Slope* | *Intercept* | *FID* | *AD* |
| --- | --- | --- | --- | --- |
| *Gymnorhina tibicen* | 1.42 | 13.25 | 15.60 | 50.09 |
| *Pelecanus conspicillatus* | 0.11 | 102.90 | 103.14 | 62.71 |
| *Tadorna tadornoides* | 8.55 | 111.05 | 128.39 | 55.42 |
| *Threskiornis molucca* | 6.81 | 39.67 | 54.92 | 56.84 |
| *Chenonetta jubata* | 4.17 | 7.48 | 14.40 | 53.27 |
| *Cygnus atratus* | -18.50 | 99.10 | 61.56 | 63.71 |
| *Molothrus ater* | 0.02 | 26.14 | 30.33 | 44.650* |
| *Cereopsis novaehollandiae* | 32.33 | 37.33 | 92.30 | 60.97 |
| *Corvus corone* | 5.20 | -15.49 | 101.91 | 51.62 |
| *Anas castanea* | -13.30 | 97.42 | 70.44 | 52.50 |
| *Turdus merula* | 0.10 | 35.18 | 37.30 | 47.84 |
| *Phaps chalcopetra* | 2.50 | 12.25 | 17.50 | 50.49 |
| *Fringilla coelebs* | 0.57 | 3.58 | 16.30 | 45.95 |
| *Acridotheres tristis* | 5.48 | 4.84 | 12.33 | 48.13 |
| *Galerida cristata* | 6.48 | -75.70 | 23.30 | 46.66 |
| *Ocyphaps lophotes* | -0.89 | 13.88 | 12.67 | 49.27 |
| *Ardea modesta* | -8.05 | 69.71 | 52.00 | 54.69 |
| *Platycercus eximius* | 0.50 | 25.45 | 26.50 | 48.01 |
| *Streptopelia decaocto* | 1.84 | -5.98 | 23.40 | 48.80 |
| *Fulica atra* | -2.46 | 77.20 | 71.68 | 53.60 |
| *Pica pica* | -3.46 | 127.50 | 55.50 | 49.63 |
| *Carduelis carduelis* | -0.01 | 5.73 | 5.55 | 13.700* |
| *Sturnus vulgaris* | 1.72 | 25.92 | 43.56 | 47.61 |
| *Cacatua roseicapilla* | 2.00 | 8.80 | 13.00 | 50.45 |
| *Phalacrocorax carbo* | -37.46 | 128.40 | 58.47 | 59.35 |
| *Anas gracilis* | -7.60 | 92.45 | 76.64 | 51.68 |
| *Bostrychia hagedash* | 2.83 | 4.27 | 7.95 | 9.900* |
| *Aythya australis* | -18.00 | 113.00 | 75.20 | 53.21 |
| *Passer domesticus* | 1.53 | 3.00 | 23.69 | 12.500* |
| *Phalacrocorax sulcirostris* | -18.10 | 86.78 | 52.40 | 53.43 |
| *Microcarbo melanoleucos* | -8.40 | 63.80 | 46.76 | 53.00 |
| *Corvus mellori* | -2.82 | 20.52 | 16.67 | 51.70 |
| *Anthochaera chrysoptera* | -0.50 | 12.55 | 11.50 | 47.23 |
| *Grallina cyanoleuca* | 5.58 | 7.54 | 16.80 | 47.77 |
| *Vanellus miles* | 5.83 | 29.29 | 40.07 | 49.73 |
| *Biziura lobata* | -18.82 | 89.10 | 49.58 | 57.10 |
| *Manorina melanocephala* | 1.24 | 6.53 | 8.60 | 47.25 |
| *Anas superciliosa* | -22.09 | 115.55 | 66.95 | 53.90 |
| *Phalacrocorax varius* | -13.20 | 107.42 | 82.35 | 56.33 |
| *Columba livia* | 0.05 | 11.65 | 14.37 | 50.65 |
| *Malacorhynchus membranaceus* | -23.42 | 107.00 | 66.72 | 50.81 |
| *Porphyrio porphyrio* | 5.67 | 33.46 | 43.83 | 52.36 |
| *Platalea regia* | -2.46 | 52.90 | 47.73 | 56.29 |
| *Larus novaehollandiae* | 5.04 | 6.05 | 17.80 | 50.32 |
| *Threskiornis spinicollis* | -11.86 | 114.40 | 89.50 | 55.02 |
| *Malurus cyaneus* | 1.45 | 7.02 | 9.00 | 45.32 |
| *Cathartes aura* | 3.76 | 38.78 | 101.47 | 56.96 |
| *Egretta novaehollandiae* | 12.04 | 29.30 | 51.77 | 51.77 |
| *Rhipidura leucophrys* | -0.65 | 11.55 | 10.67 | 45.86 |
| *Columba palumbus* | 1.03 | 9.59 | 28.58 | 51.54 |
