# Supplementary material for: Can we use antipredator behavior theory to predict wildlife responses to high-speed vehicles?
Source: PLoS One. 2022 May 12;17(5):e0267774. doi: 10.1371/journal.pone.0267774 (PMC9098083; doi:10.1371/journal.pone.0267774)

S4: *Spatial Margin of Safety Simulation*

Figure S.10. a) The mean *p-value* across 500 different linear regression between FID and approach speed when the slope of the simulated FID data ranged from ranging from -1 to 1 in intervals of 0.05. The red line represents the significance threshold *p-value* of 0.05. b) The mean *p-value* across 500 iterations from a linear regression between FID and approach speed when the slope of the simulated FID data ranged from ranging from -10 to 10 in intervals of 0.5.

Both figure S.10 a & b suggests the range of a spatial margin of safety, that is when the slope of the FID and AD relationship is not significantly different from 0, is between -0.1 and 0.1. Figure A.2 b suggest that FID becomes invariant with approach speed when the slope of the FID and approach speed relationship is either less than -1.5 or greater then 1. A spatial margin of safety is only when the slope is near zero and non-significant. The significance of the relationship between FID and approach is not relevant for a temporal margin of safety or a delayed margin of safety. A temporal margin of safety occurs when the slope is positive and a delayed margin of safety occurs when the slope is negative. The insignificant relationship between FID and approach speed at more extreme slope values occurs because given the rapid increase in approach speed (i.e., 1 m/s to 100 m/s) results in FID reaching the lower or upper limit respectively either 0 or AD. These limitations on FID are fundamentally different from the spatial margin of safety which is a behavioral rule the animal may follow.

b)

a)


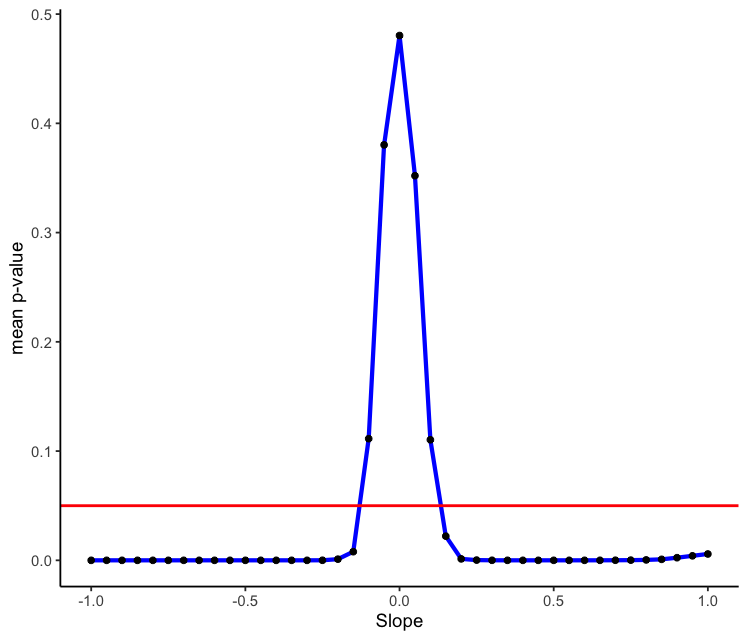

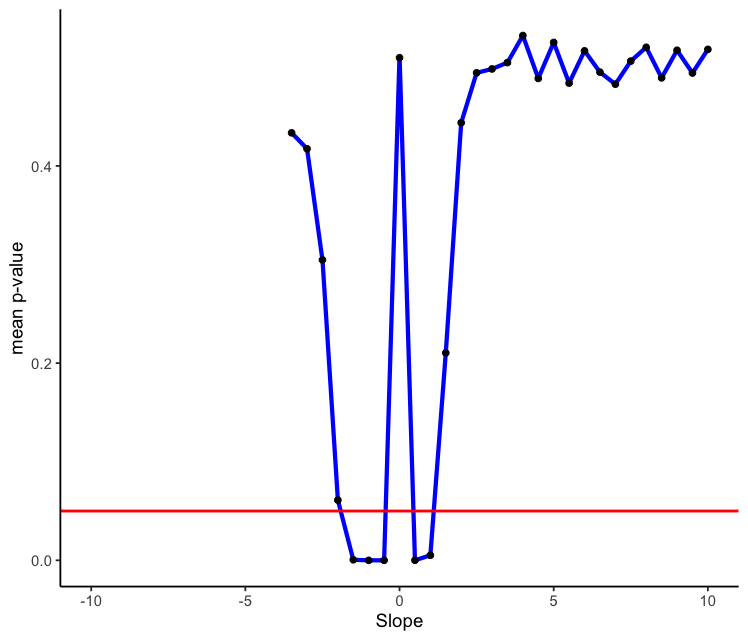

Supplement: S4 Appendix — (DOCX) [file pone.0267774.s004.docx]
