# Supplementary material for: Can we use antipredator behavior theory to predict wildlife responses to high-speed vehicles?
Source: PLoS One. 2022 May 12;17(5):e0267774. doi: 10.1371/journal.pone.0267774 (PMC9098083; doi:10.1371/journal.pone.0267774)

S5: *Discussion on the application of critical vehicle approach speed*

Figure S.11. The graph demonstrates the application of the critical vehicle approach speeds (abbreviated CVAS) for species with different spatial margins of safety, that is different FIDs.


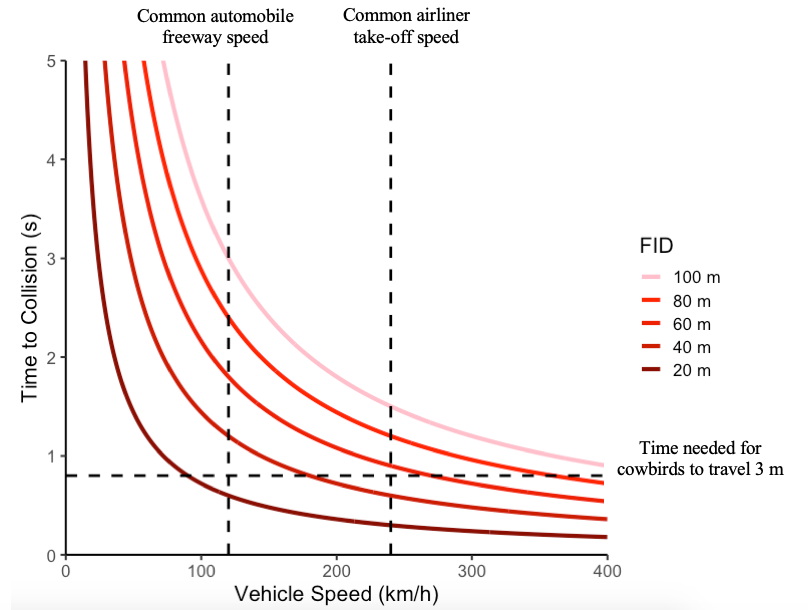

Supplement: S5 Appendix — (DOCX) [file pone.0267774.s005.docx]
