# Supplementary material for: Can we use antipredator behavior theory to predict wildlife responses to high-speed vehicles?
Source: PLoS One. 2022 May 12;17(5):e0267774. doi: 10.1371/journal.pone.0267774 (PMC9098083; doi:10.1371/journal.pone.0267774)
Supplement: S6 Appendix — (DOCX) [file pone.0267774.s006.docx]

**S6 Appendix. Code used to generate the quantitative predictions for each model of escape behavior and for simulation used to evaluate sensitivity to approach speed in the model.**

[**https://osf.io/b4cs2/?view_only=94c33be8a061462399c81dace7884151**](https://osf.io/b4cs2/?view_only=94c33be8a061462399c81dace7884151)
